# Effects of Railroad Travel upon the Health of Women

**Published:** 1872-04

**Authors:** Ely Van De Warker

**Affiliations:** Syracuse, New York


THE GEORGIA
MEDICAL COMPANION:
A MONTHLY ADVISER.

VOL. 2. ATLANTA, GA., APRIL, 1872. NO. 4

### PART I.

### Original Communications and Special Selections.

### EFFECTS OF RAILROAD TRAVEL UPON THE HEALTH OF WOMEN.

BY ELY VAN DE WARKER, M. D., SYRACUSE, NEW YORK.

Those of us who have carefully observed the passengers alight from the through trains, must have been struck by an invariable appearance. The women who have traveled long distances are haggard and weary. They accept the attention of their escort in a helpless, grateful way, more thankful than words.

When a woman has reached the end of a long railroad journey, if she has her own way, she goes promptly to bed. From one night to twenty-four hours' of continued rest is required to restore the brilliancy to her eyes and the bloom to her cheeks.

Women are very susceptible to nervous shock; and railroad traveling is peculiarly adapted to induce nervous shock. Nor is this state of nervous exhaustion confined to women. Men of nervous temperament, and others of small powers of endurance, suffer extremely after twenty-four hours or so of railroad travel. The United States, from its lines of railroad communication between commercial centres widely separated, and from the vast passenger traffic upon the great through arteries of travel, offers great facilities for the development of the nervous affections characteristic of this mode of transit. Not only is the length of the journey a factor in the production of nervous exhaustion, but another and less unavoidable condition ranks high among the causes. This is the imperfect construction of American railroads. In the United States the injurious effects of railroad travel have been imperfectly studied; but I believe such effects are developed in excess. Even in England, where the cost of railroads by the mile, greatly exceeds that of this country, the effects of rail travel upon the health has been long observed. In fact, the literature of this subject is almost exclusively English.

Mr. W. Bridges Adams, the eminent railway engineer, is explicit on the various mechanical causes of the injurious influences on health. These causes are inseparable from rail travel, even on the best constructed roads.

It will save pages of description to tabulate Mr. Adams' mechanical analysis of movements in railroad carriages when in motion:

*Table of Causes of Movements of Railroad Carriages When in Motion.*

| | | |
|---|---|---|
| The Wheels. | The Vibration. | Common to all vehicles.<br>In R. R. carriages: Rigidity of springs.<br>Regular rough surface to roll over; resulting in incessant rhythmical, short vibrations and a humming sound. |
| | The Oscillation. | The wheels taking the course of the least resistance.<br>"Sledging" of the wheels upon the rails, increasing with velocity.<br>The carriage moving not on wheels, but on rollers. |
| The Rails. | The hammer sound. | The flanges striking the rails from side to side.<br>Want of perfect rolling movement. |
| | Irregular jolting. | The rails flatten out irregularly.<br>Wheels wear in grooves.<br>The wearing of "chairs."<br>The "fishing" of joints work loose. |
| | Regular jolting. | The working loose of the chairs on the ties.<br>Defective joints.<br>The tilting of rails, loose at the joints, by the passing of trucks. |

On a new road, on an old or badly constructed road all these causes of the movements and sounds in running passenger carriages are intensified. Mr. Adams' account* is fairly written, and if this is the condition of things existing in England, how more marked must the same conditions be on American railroads. Not only have we the incessant and varied motion and sound as a cause of nerve exhaustion; but the very condition to make this exhaustion the more easily felt is the faulty ventilation of passenger coaches. No other cause so lays the human system open to the assaults of disease, or derangement in nerve function as the respiration of impure air. On improved coaches it seems as if every effort was made to improve ventilation: but in sleeping coaches, from their internal arrangements, it is impossible to supply the necessary amount of pure air. It is upon the great through routes of travel, on which thousands of women spend from one to two or more days and nights, that motion, sound, and pestilential atmosphere produce their most injurious results.

Women are poor travellers, the world over. From the time her trunk is packed until her journey is over, she is in a state of cruel excitement. Nor is this state confined to women. Men are comparatively common in whom this anxiety and agitation practically incapacitated them from habitual travelling. Dr. Brown Sequard observes† that this must prove injurious. In certain unhealthy conditions of the heart it has proved fatal.

The effect on the muscular system of the short rapid vibrations and oscillations is that of calling into a state of constant relaxation and contraction a large number of muscles, especially those which act upon the pelvis to maintain an erect position, and the muscles of the neck to prevent the head from rolling. There is also a constant bracing of the feet to counteract the roll of the coach.

The effect upon the nervous system is as well marked. Without going into any examination of the pathology of this, let us see what railroad travelling often does. The traveller—and in this case it is generally a woman—becomes nauseated or vomits. The

---

*London Lancet, I., 1862, 337 Amer. Ed.
†London Lancet, loc. cit.

explanation of this phenomenon becomes a type of the effects upon the system at large. Dr. Brown-Sequard, in his letter to the Commission "on the Influence of Rail-way travelling on the Public Health,"* explains it by the influence of a rail-way carriage upon the phrenic, splanchnic, and vagus nerves. Irritation of the vagus nerve produces vomiting. A second cause, as proved by the experiment of Luschka, is the irritation of the phrenic nerve by the shaking of the diaphragm. He shows that the faintness accompanying the nausea of rail travelling is due to irritation of the sympathetic nerve in the abdomen, which induces cardiac syncope by reflex action through the impressions conveyed by the splanchnic nerves acting upon the vagus through the spinal cord and medulla oblongata.†

Taking the above as one of the nervous deviations from the normal incident to rail travel; we have just reasons for searching for others less evident but equally profound, or for explaining many obscure lesions of the nervous system in this manner.

It is not uncommon to read in the local columns of newspapers of the delivery of pregnant women, either on trains, or in passenger waiting-rooms. Not only are these labors short of the standard duration of pregnancy, but they are frequently abortions. It is common to hear women say, who have suffered from menstrual irregularity, that they came around while on the cars. I have treated several women under these circumstances, and I have strong reasons for believing that in several ovular abortions have occurred. I cite these instances to show that other morbid changes may be induced, the result, probably, of combined mechanical causes, and of nervous shock. Nearly all the prominent symptoms of rail travel exhaustion and sickness are direct results of its injurious effects upon the great nerve centres.

Since I became interested in the diseases peculiar to women, I have never ceased to regard her as an anomoly in Nature. She is a womb-bearing animal standing erect. It is throwing one more fact in the way of Darwinism. Comparing her pelvis with that of lower animals and it is, as it were, insufficient in its change of

---

*London Lancet, loc. ci

†London Lancet, II., 18 2, 324. A. E.

type with her change in sphere. With a womb that is pendulous in a rigid cavity. An organ that sustains a burden of viscera above, and subject to a potent periodical function. This in a creature prone to sexual emotion, which, among the married, is subject to a facility of gratification from which all other animals recoil; and it follows as a natural sequence that many positions in which she is placed react with dangerous violence.

The action of railroad travel upon the sexual apparatus of woman is marked. This is particularly the case if these organs are suffering from previous derangement. Under these circumstances no form of womb disease exists but what are aggravated; no form of functional deviations from the normal but what are intensified. This applies forcibly to the menstrual function. Dysmenorrhœal pains when the discharge makes its appearance during or immediately after a fatiguing railroad journey are almost invariably severe in their character, and attended with unwonted prostration. This condition may exist so intensely, that, at these times a dysmenorrhœal woman is incapacitated from travelling, although able to attend to household duties. The following case will illustrate this:

CASE 1. Miss Ella T., æt. 18, a well developed blonde, commuted over the Troy and Boston Railroad for fare from a point fifteen miles from Troy each day, and return, for the purpose of attending a young ladies' seminary in that city. I was called to see her in October, 1867, at a hotel, where she was obliged to go, being unable to reach her school. The night before she began to menstruate, with the amount of pain habitual with her. Her rule was to remain at home during the first three days of menstruation, knowing too well the effect her daily railroad journey had upon her at that time. The attack began with intense expulsive pains when near the city. Pain darting down the thighs; backache, and a slightly increased menstrual flow. When I saw her, half an hour after her arrival, there was intense pain as above. The face was pale and the body cold. The pulse was thready. Great restlessness, throwing herself violently about, tenderness on pressure over the hypogastrium. After I saw her she vomited, and from the appearance of the gastric contents, her breakfast had not digested. She was unwilling and unable to attempt the brief journey home the next day by rail, but enjoyed a carriage drive. She was confident, from her past experience, that the journey home by rail would have resulted in a second attack.

It is not difficult to make a practical application of the above case to thousands placed in the same situation. The numerous lines of railroad converging in the large cities take to the schools each morning scores of young girls just entering the most trying period of menstrual life. From the moment their eyes are open in the morning, until seated in the early train, they are in a state of agitation and excitement. A hasty and insufficient breakfast is taken, in constant fear of losing the train. While seated there she is giving the last glance at her lessons. A day of study, a cold lunch, and a weary journey home again, completes a day of as miserable existence as there is on earth. What effect such a life at that critical epoch of womanhood will have upon her future usefulness, it does not need imagination to depict.

The ovaries come in for their share of the wear and tear of the organs due to this mode of transit. They from an anatomical necessity, are involved. From their comparatively helpless position on the ilia, and from their firm connection with the womb, which is in constant motion with each movement of the train, ovarian pain from a slight sense of uneasiness to a degree of pain which is truly appalling is of common occurrence. The following case will well illustrate this form of ovarian pain :

Case 2. Mrs. T., æt. 31, a well developed and muscular woman, the mother of two children, the youngest four years old, rode on the 18th of March, 1867, from New York to Troy on the Hudson River Railroad. She was attacked by pain in the right ovarian region, which gradually grew worse, so that on reaching her destination it was excruciating in its intensity. When I saw her some hours after, there was tenderness on pressure in the right iliac region, and a sense of heat and throbbing. She gave a peculiar history of her different railroad journeys. Three times before she had experienced similar attacks, but never on so short a road before. Usually, after some hours' ride the pain, generally on the right side, would come on, and in the course of a few hours would reach its climax of intensity.

CASE 3. Miss H. A., æt. 24, a small and vivacious brunette—a book agent. Travelled frequently long and short distances on the New York Central and Hudson River Railroad—one of the best constructed roads in the country—and in the last few months had suffered from constant pain in both groins, greatly aggravated by her journeys from place to place. Menstruation had grown scanty and painful, with a profuse leucorrhœa during a great share of the interval. Pain in the back was also present, but her great source of annoyance was a dull, throbbing pain in both iliac regions. She had waited until she had began to fail in appetite and strength before she sought medical advice. She refused positively to submit to an examination. Bromide of potass. gave great relief, and with no other treatment, excepc to abstain from her railroad life, she was restored to her usual health in about two months.

These cases I believe to be typical of a class by no means uncommon. The fact that it is not common to have our attention called to them is no evidence of their rarity; for women are famous as silent sufferers when the sexual organs are the seat of the derangement. The causes which produce this ovarian irritation are undoubtedly mechanical and have their source in the constant and unyielding forging of truck wheels of passenger coaches.

Another accident to which a class of women are exposed while on railroad journeys is premature labor, to which I have already briefly referred. Its importance demands a more extended notice. From the evidence of railroad employees, I believe this accident is more common than is generally supposed—especially upon emigrant trains. It is also quite a common news item in city papers as occurring upon a train or in the waiting rooms. Among the thousands of married women travelling there must be many who have reached the limit of utero-gestation; but I do not wish to include this class. The premature labor and abortion are the results which I shall cite. These accidents are not common among healthy women. I presume the majority of women railroad travellers are healthy; and if that presumption is granted, it follows that the peculiar nature of the method of transit is the cause. Two cases have occurred to me, which I believe may be directly traced to this cause.

Case 4. I was called to see Mrs. J. O., æt. 28, the mother of two children, the youngest 3 years old. She had arrived on an evening train. She came direct from Toledo, Ohio, en route for New York, and was taken sick after passing Rochester. When I saw her—about three hours from the beginning of the attack—she was evidently suffering from a pending abortion. She aborted during the night of a fœtus at three months. She never had an abortion before, and gave a history of perfect health up to the date of this occurrence.

Dr. Meadows, physician for the diseases of women and children at King's College Hospital, reports the following cases to the commission:

Case 5. Mrs. P., æt. 27, pregnant at seventh month with second child (first born at full term), travelled from Ipswick to London by express—a distance of seventy miles; began to feel uncomfortable during last half hour of the journey, and was taken in labor two hours after her arrival. There was obstinate flooding before the birth of the child, much worse after, and from the effects of which she died the following morning.

Case 6. Mrs. W., æt. 34, pregnant at the tenth week, traveled by rail from Edinburgh to London. A slight "show" began while in the train. It increased after her arrival; but by the employment of astringents and absolute rest and quiet, no further mischief ensued.

Case 7. Mrs. G., aged 40, pregnant at the fourteenth week, with the seventh child, traveled by rail from Torquay to London. Pain and hemorrhage began while in the train, and in half an hour after her arrival in London ovum was expelled with considerable hemorrhage. Dr. Meadow says that at the hospital out-patients, suffering from polypus and cancer of the uterus, have frequently told him that even in coming short distances by rail, bleeding frequently followed the journey.

The conclusions are that there is greater danger to pregnant women in travelling by rail in the latter than in the earlier months of gestation. Dr. Graily Hewitt, in his report to the same commission, says: "My own experience would lead me to the conclusion that the danger of producing abortion by rail-way travelling is slight, so far as short distances are concerned, but that long journeys cannot be undertaken with impunity, or with freedom from the risk of abortion. The fatigue consequent on maintenance of a somewhat constrained and uniform position during many hours, must indirectly favor its occurrence; and shocks, however slight, when continually applied during several successive hours, would have a more directly positive effect in the same direction."

Another evidence of the tendency to uterine engorgements is the frequency with which the menses make their appearance in women who are on, or have finished a fatiguing railroad journey; not only anticipating their usual period, but in some cases beginning a long course of menstrual irregularity. A railroad journey of some length is an efficient emmenagogue, similar in kind to the horse-back exercise recommended by the old authors. In the case of the unimpregnated womb the engorgement is probably the result of reflex irritation, including the ovaries in the circle of action, as well as the uterine nerves. I believe, as a rule, that the womb, when healthy and in an unimpregnated state, is slow to respond to sudden causes. The irritation of the sexual system which results from this cause must be profound to effect such a remarkable result.

The nausea and vomiting, already explained on Dr. Brown-Sequard's theory, is more common among women than among men; sea-sickness, on the contrary—so far as my experience goes—prevails equally in the two sexes. The nausea and vomiting from rail motion comes on generally after being on a train for some hours, and grows gradually worse; the sufferer seldom gaining immunity by the acquired habitat of the moving train; while in sea-sickness the voyager generally becomes accustomed to the moving vessel. This condition of culminating intensity of ganglionic irritation implies a cause to which the nervous system cannot become accustomed.

Another symptom of functional disturbance very common among women railroad travellers is vesical disturbance. In addition to the irritation resulting from the mode of travel, the natural modesty of women prevents their answering the calls of nature in many instances, and thus lead to undue accumulation of urine. The most common form of vesical disturbance is incontinence. This is often complicated with burning pain in the act of micturition. This sometimes amounts to strangury. In one case which I saw the strangury lasted for several days. She had journeyed direct from Chicago, and commenced in neglect in emptying the bladder.

A painful affection which seems traceable directly to neurosis from this cause is an intense hypogastric pain resembling colica scortorum, so common among an unfortunate class.

Case 8. Mrs. S., aged 24, a small nervous blonde, four years married and childless, started from her home in Savannah, Ga., to visit friends near Geneva, in Central New York. She had a pleasant trip by steamer to New York, and rested one night in that city. The next day she took a Hudson River train, and before reaching Albany pain began in the hypogastric region, passing up through both groins, and radiating over the abdomen. On reaching Syracuse she experienced such pain that she was obliged to leave the train. She entered a drug store for medicine. Here I was called to see her. She did not complain of tenderness at any point over the abdomen on pressure. Her pulse was 108 and feeble; skin moist and cold. The bowels had been regular, and appetite, before the attack, good. No sickness at any time. There was present a frequent desire to urinate, with burning pain. The uterus was not tender on pressure. She was fourteen days over menstruation. In this case, from the absence of any disturbance of function, except that of the bladder, I regarded it as purely a neurosis. Morphia, used hypodermically, soon allayed the pain. She finished her journey the next day.

Soreness, and lameness of the muscles of the neck, back and abdomen and extremities, from the constant strain on the muscles of these parts, to maintain the body in a firm position on the seat, are very common in those who have finished long journeys by rail. The muscular strain leads to retarded circulation in the lower extremities. Cold and often swelling of the feet result, especially in women advanced in life. It is much more difficult to keep warm in a railroad coach than in any other vehicle equally protected.

Dr. C. J. B. Williams, in a letter to the commission several times referred to, says of the shaking motion of rail-way carriages:— The shaking motion of railway carriages are commonly most felt in the back, loins, waist and head. If the journey is prolonged, that which first excites pain and irritation may, in the end, cause faintness and exhaustion in weakly persons; and this may be followed by feverish reaction, lasting some days. From the sameness of position, as well as the muscular strain in the extremities, some nervous people, and particularly women, are subject to pricking and sleepy (engourdissement) sensations in the lower extremities, which in long journeys are a source of great annoyance.

In estimating the influence of long journeys by rail upon women, we have the corroborative evidence of the effects of the same mode of conveyance upon men, who either follow the railroad as an employment, or habitually make long or frequent journeys by rail. The literature of this subject is positive in its evidence of the injurious effects so resulting.

Besides the able and exhaustive commission report referred to, Mr. Finlaison* offers valuable statistics of the results of such employment or feeble health of those who follow it for their sustenance. English society, owing to its organized subdivision, presents peculiar facilities for such investigations.

Mr. Nelson† also contributes valuable information upon this subject, derived, in a great measure, from the same source and with reference to the same class as Mr. Finlaison.

French literature is quite rich in the same direction, and in that country has excited great feeling. Monographs have been published by Drs. Pietra Santa‡ and Devilliers§ upon the influence on the health of railroad employees, and papers have been read by Drs. Martinet Bisson and Cahen at the Academy of Sciences. Dr. Develliers, although as chief physician of the Lyons and Paris

*Report on Friendly Societies; London. 1857.
†Contributions to Vital Statistics; London.
‡Chemins de Fer et Sante Publique; Paris: Hachette. 1861.
§Recherches Statistiques et Scientifiques sur les Maladies des Diverses Professions du Chemin de Fer de Lyons. Paris: Labe, 1857.

Railway, he investigates the subject as the avowed defender of the company, comes to important conclusions. One-fifteenth of the drivers and firemen were suffering from brain and nervous diseases, and among the brakemen and inspectors the same diseases were relatively numerous. Dr. M. H. de Martinet was so impressed by the peculiar symptoms of nerve lesion, which he found to exist among railroad employees, that he describes it as a new disease. The result of his observations is as follows*: "The persons affected grow thin; the generative power dies out; the body is agitated by startings and convulsions. The intelligence is weakened."

With all this evidence of the gravity of the functional and structural lesion on the more sturdy and robust physique of man, we have no trouble in concluding that woman—taking into consideration the less amount of exposure attending her life—must suffer in increased proportion. Since in this day of advanced ideas, when women are continually increasing their field of labor, and their opportunities for competition with more hardy manhood, it is proper to keep ever with her in her forced advance, and, if possible, before her, that she may be checked in her eager rivalry, and warned from dangerous fields. Women are travelling agents, sewing machine agents, travelling doctors and lecturers, and commercial drummers and book canvassers. It is the higher fields of labor which she invades, and not the lower. Labor which requires brain-work and nerve-work is hers by choice. The past ten years has opened for woman a great and possibly dangerous experiment. That she may be useful in these new directions of labor I do not doubt, but that each individual woman can long be successful at many of the employments now followed by her, I do not believe. Woman cannot, from the nature of her organization, make a successful habitual railroad traveller. Whatever seeming success she may achieve at an occupation which requires that mode of transit, is at the expense of her future usefulness, and for which the penalty will be pain and suffering. I believe that many women who are thus earning their daily bread, are now paying the penalty which has been measured out to them in pain. If it may not be

*Academie du Sciences, Seance du 23 Feorier.

woman's forte to do all that men can do, it is her forte to suffer silently with a heroism man never equalled.

The practical application of this paper would be in aiding the physician in the solution of the difficulty which often hampers him when his opinion is asked as to the propriety of a journey by rail for a patient. In the knowledge of the injurious effects of long railroad rides upon women even in a state of health, we must exercise considerable caution in giving our consent to such an undertaking by one who is suffering or convalescent from any sickness. When the sexual apparatus is the seat of disease, it would be the wisest to advise a postponement of the journey until such time as the organs may be in a state of health; or, in case of necessity, to put her in as good shape as possible for the trip. For women who are pregnant it is necessary to exercise still more care in the expression of our opinion; and in all cases strongly advise against such exposure to danger after the sixth month. In case of women who have frequently aborted, they ought to undertake such dangers to gestation on their own responsibility, without the consent of the physician, who should give an unfavorable opinion. Patients suffering from diseases of the heart, or nervous system, are equally exposed to danger in journeys by rail. In cases of heart disease death has been known to occur while in the cars in persons who, when proper care was exercised, were enjoying a comparative degree of comfort. The confusion, noise and motion, combine to render such journeys extremely wearing to nervous woman. In the matter of aged women, we ought also to express an unfavorable opinion. The agitation alone which attends unwonted scenes and commotion, would be sufficient to render such an undertaking dangerous to one so sensitive to impressions as an aged woman.

In 1825 there was but one railway carriage in the world. It bore upon its unsightly, yellow sides the motto:

"*Periculum privatum, publica utilitas.*"

The people living on the line of this embryo railroad—the progenitor of such mighty changes in men's lives—did not believe the two first words of this motto, and the more this subject is studied, the clearer becomes the fact that it is not devoid of danger. But all the inventions of this fevered age which have been devised to surmount space and time, are achieved at the expense of safety and health. This is so universal that it has all the force of a law.

